# A single center analysis of first-line treatment in advanced KRAS mutant non-small cell lung cancer: real-world practice

**DOI:** 10.1186/s12885-022-10236-9

**Published:** 2022-11-14

**Authors:** Yanxia Liu, Yuan Gao, Ying Wang, Cong Zhao, Zhiyun Zhang, Baolan Li, Tongmei Zhang

**Affiliations:** 1 Medical Oncology, Tuberculosis and Thoracic Tumor Research Institute, Beijing Chest Hospital, Capital Medical University, Beijing, China; 2 Cancer Research Center, Tuberculosis and Thoracic Tumor Research Institute, Beijing Chest Hospital, Capital Medical University, Beijing, China

**Keywords:** KRAS mutation, NSCLC, First-line treatment, Immunotherapy, Antiangiogenic therapy

## Abstract

**Purpose:**

For the first-line treatment of KRAS mutant non-small cell lung cancer (NSCLC) patients, immunotherapy or platinum-based chemotherapy are the main treatment method. Here, we investigated the clinical efficacy and prognosis those two regimens as first-line treatment in real-world practice.

**Methods:**

KRAS mutant NSCLC patients received chemotherapy or immunotherapy as first-line treatment from September 2014 to March 2022 were enrolled. Clinical characteristics, treatment scheme, clinical curative effect and follow-up data of enrolled patients were collected for analysis.

**Results:**

Fifty patients received immunotherapy and 115 patients received chemotherapy were enrolled. Patients who received immunotherapy (HR = 0.350, 95%CI 0.156–0.781, *P* = 0.010), or pemetrexed-based regimen (HR = 0.486, 95%CI 0.255–0.928, *P* = 0.029), or antiangiogenic therapy (HR = 0.355, 95%CI 0.159–0.790, *P* = 0.011) were at a low risk of disease progression. And patients received antiangiogenic therapy had lower risk of death than those not (HR = 0.333, 95%CI 0.120–0.926, *P* = 0.035). Subgroup analysis revealed the immunotherapy compared to chemotherapy alone had lower risk of disease progression (HR = 0.377, 95%CI 0.166–0.856, *P* = 0.020) in PD-L1 expression ≥1% subgroup. And in non-G12C KRAS subgroup, but not in G12C KRAS subgroup, patients who received antiangiogenic therapy had lower risk of disease progression (HR = 0.254, 95%CI 0.098–0.656, *P* = 0.005) and death than those not (HR = 0.197, 95%CI 0.056–0.692, *P* = 0.011). In terms of different chemotherapy regimen, platinum-paclitaxel combined with antiangiogenic therapy achieved the highest ORR and DCR (*P* < 0.05), while the platinum-pemetrexed combined with antiangiogenic therapy had the longest PFS and OS (*P* < 0.001).

**Conclusion:**

For the first-line treatment of KRAS mutant NSCLC patients, immunotherapy, antiangiogenic therapy, and pemetrexed-based regimen could obtain more benefits. Subgroup analysis revealed the benefits of immunotherapy compared to chemotherapy were applicable in PD-L1 expression≥1% subgroup, and antiangiogenic therapy could benefit non-G12C KRAS subgroup, but not G12C KRAS subgroup. In terms of different chemotherapy regimen, platinum-pemetrexed combined with antiangiogenic therapy may be the preferred chemotherapy regimen.

## Introduction

Lung cancer has the highest mortality among solid tumors worldwide, and non-small cell lung cancer (NSCLC) accounts for 80–85% of all lung cancer [1]. Kirsten rat sarcoma viral oncogene homolog (KRAS) mutation is one of the most common oncogenic mutation detected in patients with NSCLC [2, 3]. KRAS gene is a member of the RAS gene family which plays an important role in regulating cellular proliferation, differentiation, and apoptosis [4]. It has been recognized that oncogenic mutations in KRAS gene result in hyper-activation of downstream signaling cascades that lead to uncontrolled cell proliferation and survival, so as to tumorgenesis [5, 6]. However, due to the special structure of KRAS protein and the wide biological functions of the gene, the development of targeted therapy for KRAS mutant lung cancer has been frustrated for many years. Recently, clinical studies of targeted drugs for KRAS G12C have made some progress [7–9]. In 2021, the US Food and Drug Administration (FDA) approved the first KRAS-targeted drug, Sotorasib, for the treatment of NSCLC patients with KRAS G12C mutations who had received at least one previous systemic therapy [10]. As for the first-line treatment of KRAS mutant NSCLC patients, immunotherapy or platinum-based chemotherapy are still the the main treatment method. Studies have revealed that intratumoral PD-L1 expression, tumor mutation burden (TMB), the intensity of CD8+ T cell infiltrates could be biomarkers of response to immunotherapy [11–13]. And among these biomarkers, PD-L1 expression is the most widely used in NSCLC patients and help to guide the selection of immunotherapy regimens. Based on the results of clinical trials, immunotherapy monotherapy has been approved as the first-line treatment for advanced epidermal growth factor receptor (EGFR)/anaplastic lymphoma kinase (ALK) negative NSCLC patients with PD-L1 expression ≥50%, and for patients with PD-L1 expression < 50%, immunotherapy combined with chemotherapy and/or antiangiogenesis therapy can bring more survival benefits than chemotherapy [14–16]. However, the results of clinical trials are difficult to reproduce in the actual clinical environment due to the strict selection of subjects and control of clinical treatment regimen, and the efficacy of real-world patients needs to be answered with real-world data. In addition, studies have found that KRAS mutant NSCLC populations are highly heterogeneous, and different subtypes and co-mutations can affect the biological characteristics and treatment response of tumors [17–20]. Therefore, how to rationally select the existing immunotherapy or chemotherapy regimen to improve the survival and life quality of KRAS mutant NSCLC patients in different subgroups is particularly important. In this study, we retrospectively analyzed the real-world clinical data of advanced KRAS mutant NSCLC patients who received immunotherapy or chemotherapy regimen as first-line treatment, and explored the efficacy and prognosis of patients in different subgroups after diverse treatment regimen, so as to provide some clues for selecting appropriate treatment regimen as first-line therapy for advanced KRAS mutant NSCLC patients in real world.

## Materials and methods

### Patients and clinical data

We retrospectively studied NSCLC patients who diagnosed in Beijing Chest Hospital, Capital Medical University from September 2014 to March 2022, and included patients with KRAS mutation who received immunotherapy or chemotherapy regimen as first-line treatment for further analysis according to inclusion and exclusion criteria.

Inclusion criteria: (1) Patients with newly diagnosed metastatic or postoperative recurrence of NSCLC diagnosed by histology or cellular sediment embedding; (2) Cytological sediment or histological samples of patients underwent genomic testing and found at least one KRAS mutation; (3) Stage IIIB to IV according to the eighth edition of the American Joint Committee on Cancer/International Union Against Cancer TNM stage classification for lung cancer; (4) Patients receiving immunotherapy or chemotherapy as first-line treatment; (5) Patients with detailed clinical treatment and prognosis information.

Exclusion criteria: (1) Patients without clear pathological diagnosis information; (2) Patients with operable NSCLC; (3) Patients with serious dysfunction of important organs; (4) Patients with other tumors at the same time or patients with other tumors in the past 5 years; (5) Patients with incomplete clinical data.

Clinical and follow-up data of the patients were collected and recorded for analysis included age, sex, pathological type, smoking history, KRAS mutation status, mutation subtypes and other associated mutations (co-mutations), performance status (PS) score, TNM stage, treatment history, clinical efficacy and prognosis information.

### Efficacy evaluation and prognosis

According to the Response Evaluation Criteria in Solid Tumors (RECIST) guideline version 1.1, the short-term efficacy was divided into complete Response (CR) and partial Response (PR), stable disease (SD) and progressive disease (PD). Objective response rate (ORR) = (CR + PR)/(CR + PR + SD + PD) × 100%; Disease control rate (DCR) = (CR + PR + SD)/(CR + PR + SD + PD) × 100%; Progression-free survival (PFS) was defined as the time from the initiation of the first-line chemotherapy until date of progression or last follow-up or death caused by any cause. Overall survival (OS) was defined as the time of postoperative disease recurrence in patients with early surgery or from the time of initial diagnosis in patients with advanced to the time of death or last follow-up.

### Follow-up

Survival follow-up was conducted by telephone inquiry and medical record inquiry system. The cut-off date is June 30, 2022.

### Statistical analysis

SPSS 23.0 and GraphPad Prism 7 were used for statistical analysis and mapping. Data were presented as frequencies and percentages for categorical variables and means or medians, with standard deviation or interquartile range for continuous variables. Whitney U test was used to compare PD-L1 expression in different subgroups. Chran’s and Mantel-Haenszel statistics and Chi-square or Fisher’s exact test were used for assessing the statistical significance of categorical variables and the odds ratio (OR) with 95% confidence intervals (95% CIs). Kapla-Meier method was used for univariate analysis of OS and PFS, whereas comparisons among the subgroups were analyzed using the log rank test. Cox proportional-hazards regression model was performed for multivariate analysis and computing the statistical significance and hazard ratio (HR) with 95% CIs. Statistical significance was set at *P* < 0.05.

## Results

### Epidemiological characteristics of KRAS mutations in NSCLC patients

A total of 5621 NSCLC patients underwent KRAS mutation gene testing in our hospital between September 2014 and March 2022, of which 3024 were detected by PCR and 2597 by NGS. And 554 (PCR:282; NGS:272) patients had positive KRAS mutation, the frequency of KRAS mutations was 9.86% (554/5621). Among the 272 KRAS mutant patients tested by NGS, the prevalent co-mutations include TP53 (102/272, 37.50%), SMAD4 (15/272, 5.51%), EGFR (14/272, 5.15%), BRAF (12/272, 4.41%), MET (8/272, 2.94%), DDR2 (8/272, 2.94%), STK11 (4/272, 1.47%). We also have obtained and analyzed the TCGA data using the cBioportal Tool (http://www.cbioportal.org/) and found that the prevalent co-mutations of KRAS mutant NSCLC patients are TTN(51.19%), RYR2(45.80%), MUC16(42.78%%), CSMD3(40.18%), LRP1B(39.55%), TP53(39.38%), USH2A(38.75%), ZFHX4(37.39%), SPTA1(32.89%), FLG(30.74%).

### Clinical characteristics of enrolled patients with KRAS mutant NSCLC

**Fig. 1:**
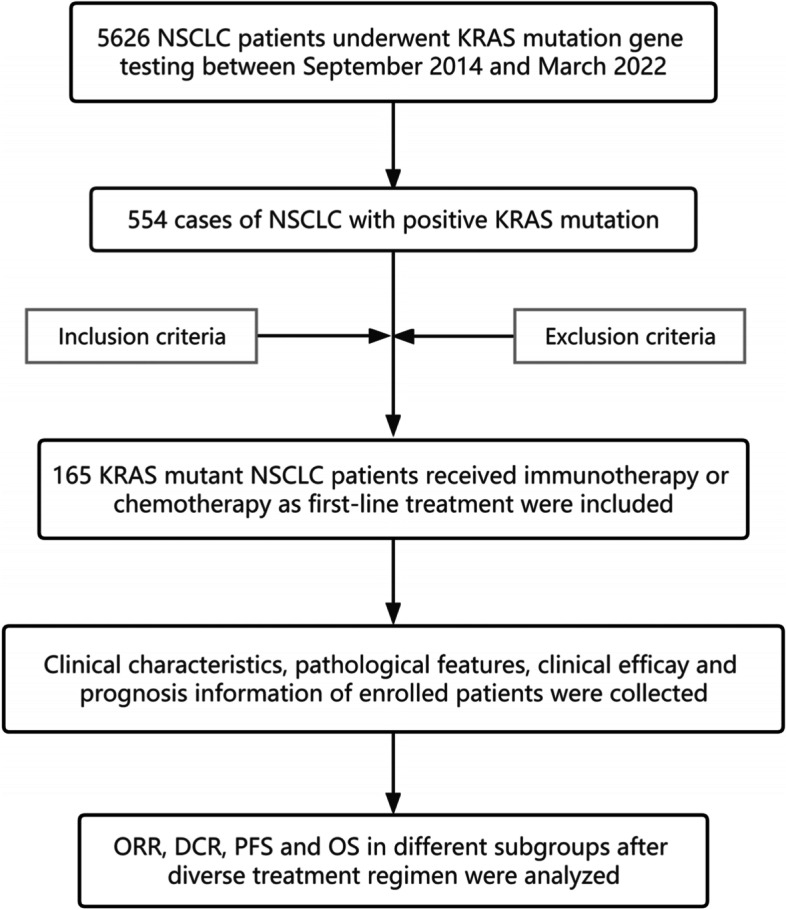
Study design and patient enrollment flowchart

**Fig. 2:**
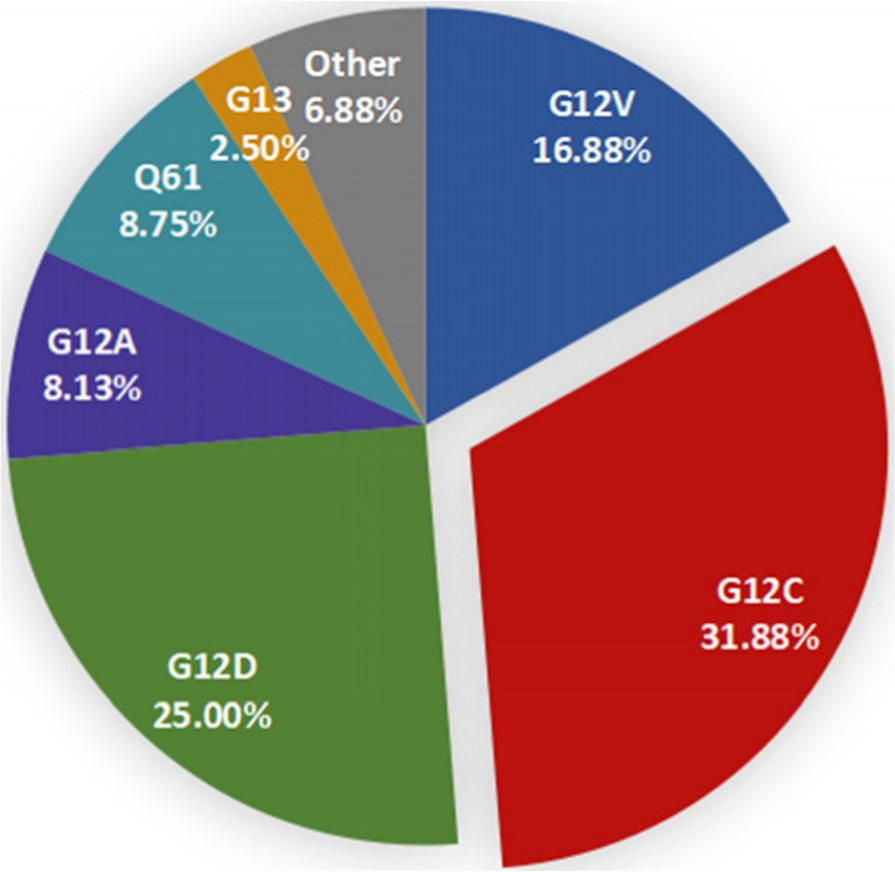
KRAS mutation subtypes in 160 NSCLC patients

**Fig. 3:**
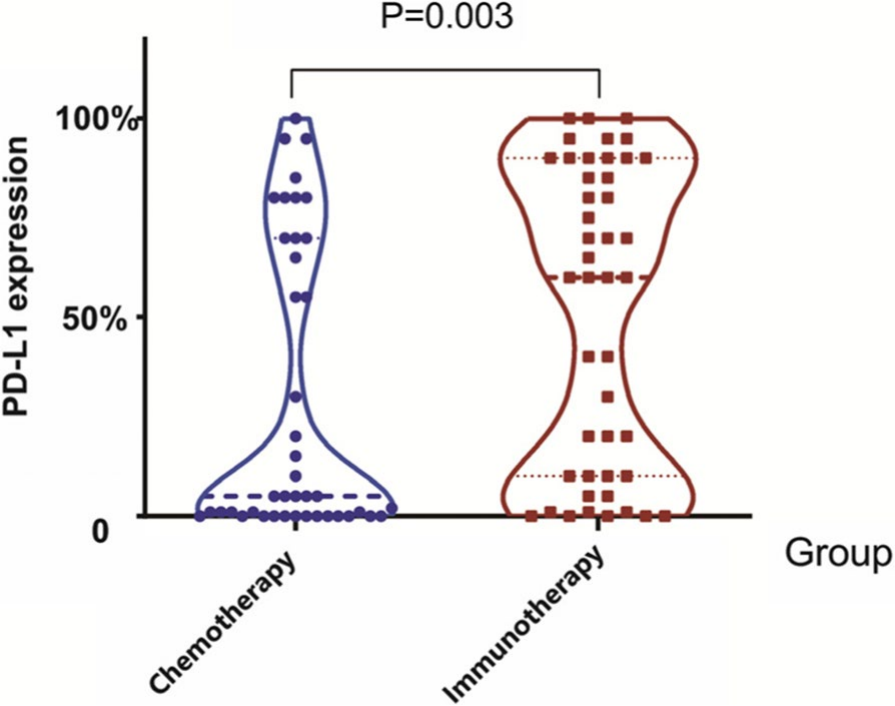
PD-L1 expression between KRAS mutant NSCLC treated with immunotherapy and chemotherapy

According to the inclusion and exclusion criteria, 115 KRAS mutant NSCLC patients received chemotherapy regimen and 65 patients received immunotherapy as first-line treatment were included, the specific process is shown in Fig. 1. The median age of 165 enrolled patients was 64.5 years (range: 36–79 years). Due to the change of genomic testing methods and kit, 81 patients detected the TP53 co-mutations. And 5 patients could only detect whether there is a KRAS mutation, but not specific to KRAS subtypes. The most common mutation sites of KRAS are located at codon 12, accounting for 82.50% (132/160), among it, KRAS G12C (51/160, 31.88%), G12V (27/160, 8.13%) and G12D (40/160, 25.00%) were the three most frequent subtypes. In addition, codon 61 accounted for 8.75% (14/160) and codon 13 accounted for 2.50% (4/160), as shown in Fig. 2. Information about PD-L1 expression was available for 87 patients. It is found that immunotherapy group has higher median PD-L1 expression (5.0% vs 60.0%, *P* = 0.003, Fig. 3) than chemotherapy group. Chi-square test was used to compare the distribution of clinical characteristic in patients treated with immunotherapy and chemotherapy, and there were no statistical differences in gender, smoking history, PS score, pathological type, Stage, while there were statistical differences in age, KRAS mutant subtypes and PD-L1 expression, as shown in Table 1.

**Table 1:** Clinical characteristics of KRAS mutant NSCLC treated with immunotherapy and chemotherapy

Baseline characteristics	Immunotherapy	Chemotherapy	*P* value
Age (year)			0.02
< 65	7(14.00%)	36(31.30%)	
≥ 65	43(86.00%)	79(68.70%)	
Sex			0.261
Male	41(82.00%)	85(73.91%)	
Female	9(18.00%)	30(26.09%)	
Smoking history			0.733
Yes	33(66.00%)	79(68.70%)	
No	17(34.00%)	36(31.30%)	
Histology			0.107
Adenocarcinoma	43(86.00%)	109(94.78%)	
Squamous	5(10.00%)	6(5.22%)	
Other	2(4.00%)	0(0.00%)	
KRAS mutant subtypes			0.003
G12C	24(48.00%)	27(24.55%)	
non-G12C	26(52.00%)	83(75.45%)	
TP53 co-mutation			0.853
positive	15(38.46%)	17(40.48%)	
negative	24(61.54%)	25(59.52%)	
PD-L1 expression			0.022
< 1%	5(11.11%)	13(30.95%)	
≥ 1%	40(88.89%)	29(69.05%)	
PS score			0.842
0–1	46(92.00%)	103(89.57%)	
= 2	4(8.00%)	12(10.43%)	
Stage			0.644
III	6(12.00%)	16(13.91%)	
IVa	26(52.00%)	66(57.39%)	
IVb	18(36.00%)	33(28.70%)	

### Treatment regimen

A total of 115 patients received chemotherapy regimen as first-line treatment were enrolled. According to whether combined antiangiogenic therapy (AT) or not, 58 (58/115, 27.1%) patients were treated with chemotherapy+antiangiogenic therapy (AT group), and 57 (57/115,72.9%) patients were treated with chemotherapy regimen alone (no-AT group). According to chemotherapy drugs, 60 (60/115, 49.5%) patients received pemetrexed combined with platinum (PEM group), 48 (48/115, 36.5%) patients received paclitaxel combined with platinum (TAX group), and 7 patients (7/115, 14.0%) received other chemotherapy drugs (OTHER group).

**Fig. 4:**
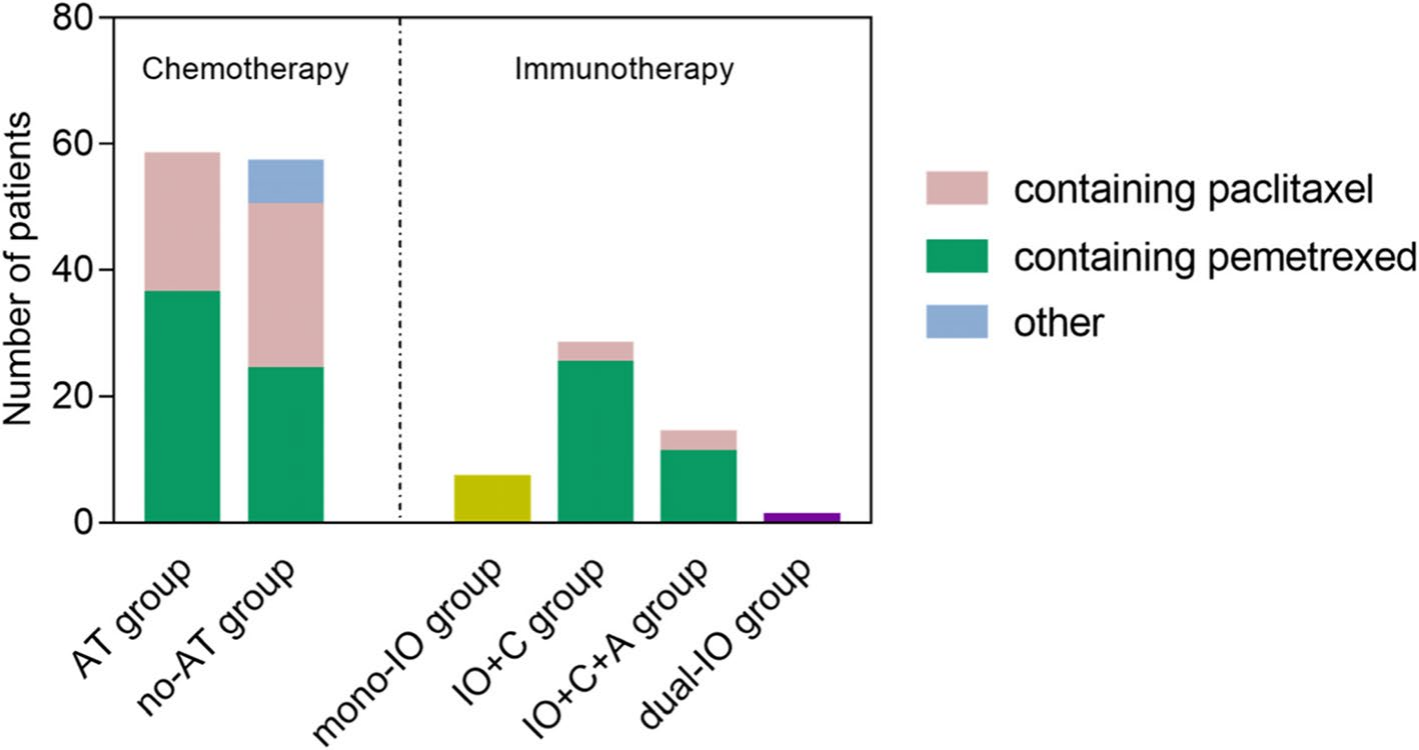
Treatment regimens of KRAS mutant NSCLC patients

Fifty patients treated with either immunotherapy monotherapy (7/50, 14.0%, mono-IO group) or combination immunotherapy. According to the combined drugs, the immune combination group can be divided into immunotherapy+chemotherapy (28/50, 56.0%, IO+C group), immunotherapy+chemotherapy+antiangiogenesis therapy (14/50, 28.0%, IO+C + A group), and dual immunotherapy (1/50, 2.0%, dual-IO group). Details were shown in Fig. 4.

### Treatment efficacy and prognosis

During the course of treatment, none of the 165 patients achieved CR, 57 patients achieved PR, and 140 patients achieved SD. The ORR and DCR of all KRAS mutant NSCLC patients were 34.55 and 84.85%, respectively. Among all KRAS mutant NSCLC, the immunotherapy group compared to chemotherapy group had higher ORR (44.00% vs 30.43%) and DCR (96.00% vs 80.00%). Considering the distribution differences of several clinical characteristics between the immunotherapy and the chemotherapy group, we used Chran’s and Mantel-Haenszel statistics to control for gender, KRAS mutation and PD-L1 expression categorical variables and found that there was no significant difference in ORR and DCR between immunotherapy and chemotherapy group. Next, we performed a subgroup analysis which also using Cochran’s and Mantel-Haenszel statistics to control for other variables, and found no statistically significant differences in ORR and DCR between the immunotherapy and the chemotherapy group in KRAS G12C subgroup, non-G12C KRAS subgroup, PD-L1 expressio≥1% subgroup, PD-L1 expression < 1% subgroup, as shown in Table 2.

**Table 2:** Treatment efficacy of KRAS mutant NSCLC received first-line treatment

KRAS mutant NSCLC	ORR			DCR		
	Chemotherapy	Immunotherapy	*P* value	Chemotherapy	Immunotherapy	*P* value
Overall	30.43% (36/165)	44.00%	0.893	80.00%	96.00%	0.439
KRAS mutant subtypes						
G12C	29.63%	54.17%	0.667	81.48%	95.83%	0.924
non-G12C	30.12%	34.62%	0.654	78.31%	92.31%	0.679
PD-L1 expression						
< 1%	15.38%	20.00%	0.686	84.62%	100.00%	0.911
≥ 1%	48.28%	47.50%	0.979	89.66%	95.00%	0.724

**Fig. 5:**
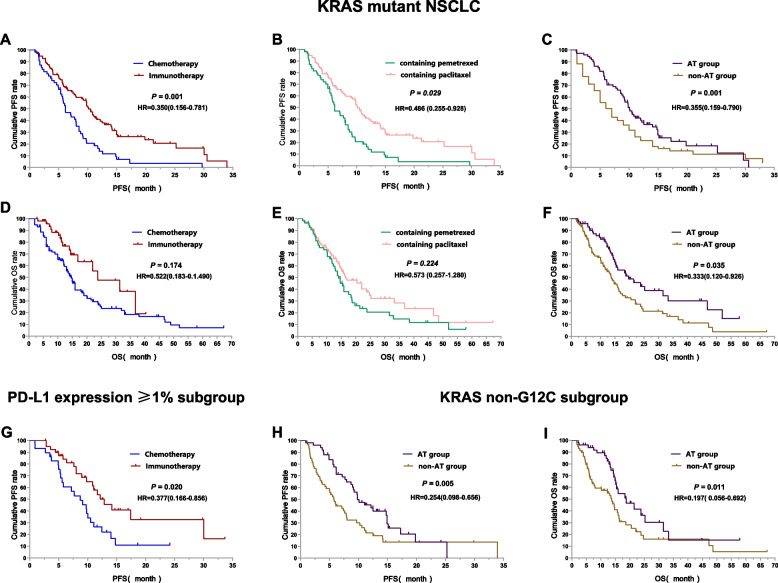
Survival curves of KRAS mutant NSCLC (**A**). The PFS survival curves between immunotherapy and chemotherapy group in KRAS mutant NSCLC patients (**B**). The PFS survival curves between treatment containing pemetrexed and containing paclitaxel in KRAS mutant NSCLC patients (**C**). The PFS survival curves between treatment with and without antiangiogenic therapy in KRAS mutant NSCLC patients (**D**). The OS survival curves between immunotherapy and chemotherapy group in KRAS mutant NSCLC patients (**E**). The OS survival curves between treatment containing pemetrexed and containing paclitaxel in KRAS mutant NSCLC patients (**F**). The OS survival curves between treatment with and without antiangiogenic therapy in KRAS mutant NSCLC patients (**G**). The PFS survival curves between immunotherapy and chemotherapy group in expression≥1% subgroup (**H**). The PFS survival curves between treatment with and without antiangiogenic therapy in non-G12C KRAS subgroup (**I**). The OS survival curves between treatment with and without antiangiogenic therapy in non-G12C KRAS subgroup

Up to the last follow-up time, a total of 126 patients had disease progression (126/165, 76.36%) and 100 patients had died (100/165, 60.61%). Median PFS and OS of all 165 KRAS mutant NSCLC were 9.0 months (95% CI 7.5–10.5) and 16.0 months (95% CI 13.3–18.7). Univariate analysis showed that age, gender, smoking history, PS score, pathological type and KRAS mutation subtype were not correlated with PFS and OS (*P* > 0.05), while various treatment scheme were correlated with PFS and OS (*P* < 0.05). Among all KRAS mutant NSCLC, the immunotherapy significantly improved PFS (11.7 vs 7.0 months, *P* < 0.001, Fig. 5A) and OS (23.8 vs 14.7 months, *P* = 0.013, Fig. 5D) compared to chemotherapy alone; Treatment containing pemetrexed had longer PFS (10.1 vs 6.2 months, *P* < 0.001, Fig. 5B) and OS (16.4 vs 14.1 months, *P* = 0.112, Fig. 5E) compared to treatment containing paclitaxel; And patients who received antiangiogenic therapy had significantly longer PFS (10.0 vs 6.5 months, *P =* 0.031, Fig. 5C) and OS (19.7 vs 13.7 months, *P* = 0.004, Fig. 5F) than those not. To control confounding factors, Cox multivariate analysis was performed on the factors with *P* < 0.2 in univariate analysis or factors considered to be related to prognosis, and the results showed that immunotherapy, chemotherapy drugs, antiangiogenic therapy were correlated with PFS and only antiangiogenic therapy was correlated with 0S. This result indicated that that patients who received immunotherapy (HR = 0.350, 95%CI 0.156–0.781, *P* = 0.010), or pemetrexed-based regimen (HR = 0.486, 95%CI 0.255–0.928, *P* = 0.029), or antiangiogenic therapy (HR = 0.355, 95%CI 0.159–0.790, *P* = 0.011) were at a low risk of disease progression. And patients received antiangiogenic therapy had lower risk of death than those not (HR = 0.333, 95%CI 0.120–0.926, *P* = 0.035), as shown in Table 3.

**Table 3:** Prognosis of KRAS mutant NSCLC received first-line treatment

Characteristics and treatment scheme	Univariate analysis		Multivariate analysis		Univariate analysis		Multivariate analysis	
	Median PFS (95%CI) (month)	*P* value	HR(95%CI)	*P* value	Median OS (95%CI)(month)	*P* value	HR(95%CI)	*P* value
Age (year)		0.504	–			0.554	–	
< 65	8.1 (5.7–10.7)				17.0 (11.8–22.1)			
≥ 65	9.8 (8.5–11.1)				16.9 (13.0–20.9)			
Sex		0.941	0.810(0.387–1.696)	0.576		0.913	0.488(0.205–1.16)	0.104
Male	9.0 (7.33–10.67)				15.7 (13.2–18.2)			
Female	8.0 (4.39–11.61)				15.9 (12.7–19.1)			
Smoking history		0.53	–			0.861	–	
Yes	8.5 (6.8–10.2)				16.0 (12.7–19.3)			
No	9.1 (5.4–12.9)				15.1 (13.0–17.3)			
Histology		0.310	–			0.973	–	
Adenocarcinoma	8.5 (6.5–10.5)				16.0 (13.2–18.8)			
Squamous	11.7 (5.7–17.7)				14.7 (7.8–21.5)			
KRAS mutant subtypes		0.306	1.204(0.634–2.282)	0.57		0.215	1.616(0.733–3.564)	0.234
G12C	10.0 (8.3–11.8)				15.4 (14.1–16.7)			
non-G12C	8.0 (5.7–10.3)				19.7 (9.9–29.5)			
PD-L1 expression		0.931	0.981(0.482–1.999)	0.959			0.841(0.363–1.949)	0.687
< 1%	9.2 (4.0–14.4)				19.7 (10.9–28.5)			
≥ 1%	10.5 (9.2–11.8)				23.8 (14.3–33.3)			
PS score		0.328	–			0.129	–	
0–1	9.0 (7.58–10.4)				16.0 (12.8–19.2)			
= 2	6.0 (5.5–6.5)				13.0 (8.6–17.4)			
Stage		0.788	–			0.582	–	
III	10.8 (5.8–15.9)				18.3 (11.4–25.2)			
IVa	9.1 (7.5–10.7)				18.0 (13.3–22.7)			
IVb	9.8 (7.6–12.0)				15.0 (12.0–18.0)			
Immunotherapy		< 0.001	0.350(0.156–0.781)	0.010		0.013	0.522(0.183–0.1.490)	0.174
Yes	11.7 (9.2–14.2)				23.8 (11.3–36.3)			
No	7.0 (5.6–8.4)				14.7 (12.3–17.1)			
Chemotherapy drugs		< 0.001	0.486 (0.255–0.928)	0.029		0.112	0.573 (0.257–1.280)	0.224
containing pemetrexed	10.1 (9.0–11.1)				16.4 (9.9–22.9)			
containing paclitaxel	6.2 (4.7–7.7)				14.1 (11.5–16.7)			
Antiangiogenic therapy		0.031	0.355(0.159–0.790)	0.011		0.004	0.333(0.120–0.926)	0.035
Yes	10.0 (8.5–11.5)				19.7 (11.8–27.6)			
No	6.5(4.7–8.4)				13.7 (11.2–16.3)			

Subgroup analysis revealed the immunotherapy compared to chemotherapy alone had an improved PFS (12.9 vs 9.0 months, *P* = 0.011, Fig. 5G) and low risk of disease progression (HR = 0.377, 95%CI 0.166–0.856, *P* = 0.020) in PD-L1 expression ≥1% subgroup, And in non-G12C KRAS subgroup, but not in G12C KRAS subgroup, patients who received antiangiogenic therapy had significantly longer PFS (7.9 vs 5.8 months, *P =* 0.007, Fig. 5H) and OS (18.7 vs 13.7 months, *P* = 0.011, Fig. 5I) and low risk of disease progression (HR = 0.254, 95%CI 0.098–0.656, *P* = 0.005) and death than those not (HR = 0.197, 95%CI 0.056–0.692, *P* = 0.011)..

### Treatment efficacy and prognosis of first-line chemotherapy

**Fig. 6:**
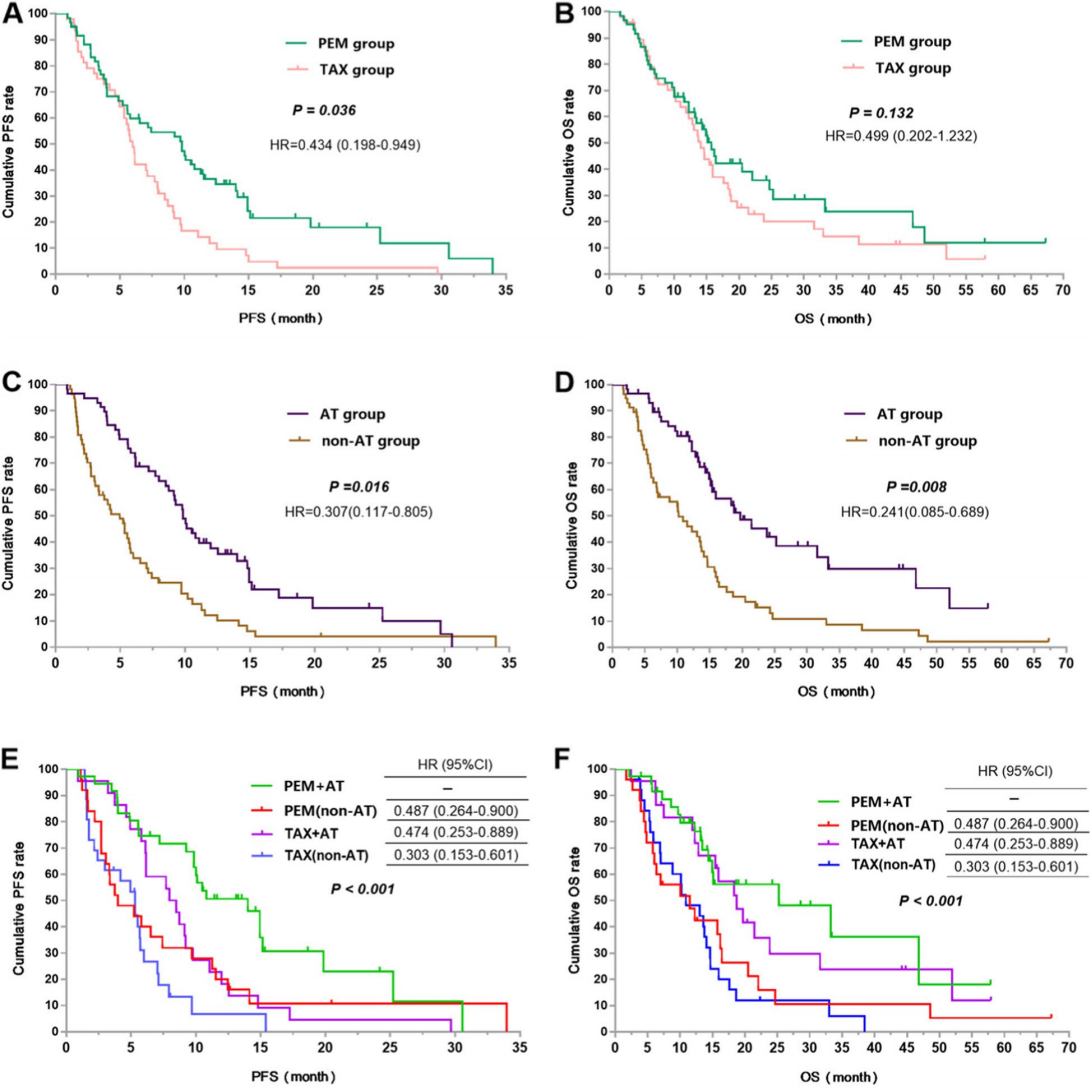
Survival curves of KRAS mutant NSCLC treated with chemotherapy (**A**). The PFS survival curves between the PEM and TAX group (**B**). The OS survival curves between the PEM and TAX group (**C**). The PFS survival curves between the AT and non-AT group (**D**). The OS survival curves between the AT and non-AT group (**E**). The PFS survival curves of different chemotherapy scheme (**F**). The OS survival curves of different chemotherapy scheme

We further analyzed the treatment efficacy and prognosis of 115 KRAS mutant patients receiving different chemotherapy regimen. Univariate analysis showed that PEM group had longer PFS (9.8 vs 6.0 months, *P* = 0.041, Fig. 6A) and OS (16.0 vs 14.0 months, *P* = 0.276, Fig. 6B). compared to TAX group. Moreover, the AT group improved PFS (9.8 vs 5.0 months, *P* < 0.001, Fig. 6C) and OS (20.0 vs 10.0 months, *P* < 0.001, Fig. 6D) in KRAS mutant NSCLC patients. Multivariate analysis revealed that patients who received pemetrexed-based regimen (HR = 0.434, 95%CI (0.198–0.949, *P* = 0.036), or antiangiogenic therapy (HR = 0.307, 95%CI 0.117–0.805, *P* = 0.016) were at a low risk of disease progression. And patients received antiangiogenic therapy had lower risk of death than those not HR = 0.241, 95%CI 0.085–0.689, *P* = 0.008), as shown in Table 4.

**Table 4:** Prognosis of KRAS mutant NSCLC received chemotherapy treatment

Characteristics and treatment scheme	Univariate analysis		Multivariate analysis		Univariate analysis		Multivariate analysis	
	Median PFS (95%CI) (month)	*P* value	HR(95%CI)	*P* value	Median OS (95%CI)(month)	*P* value	HR(95%CI)	*P* value
Age (year)		0.61	–			0.414	–	
< 65	8.7 (6.9–10.8)				14.6 (9.7–19.6)			
≥ 65	9.1 (6.5–11.7)				15.9 (14.6–17.2)			
Sex		0.337	0.810(0.387–1.696)	0.576		0.544	0.488(0.205–1.16)	0.104
Male (n=)	7.0 (5.5–8.5)				14.0 (11.7–16.3)			
Female	9.0 (4.1–13.9)				15.9 (13.7–18.2)			
Smoking history		0.847	–			0.843	–	
Yes	7.0 (5.4–8.6)				15.0 (12.2–17.8)			
No	6.0 (1.4–10.6)				13.6 (10.8–16.4)			
Histology		0.607	–			0.960	–	
Adenocarcinoma	9.4 (7.8–11.1)				14.3 (11.8–16.9)			
Squamous	10.7 (5.0–16.5)				14.6 (0–34.8)			
KRAS mutant subtypes		0.746	1.765(0791–3.939)	0.165		0.510	2.110 (0.808–5.506)	0.127
G12C	7.0 (3.2–10.8)				13.6 (4.0–23.2)			
non-G12C	7.0 (5.4–8.6)				15.0 (13.0–17.0)			
PD-L1 expression		0.271	1.027(0.417–2.526)	0.954		–	0.515(0.168–1.580)	0.246
< 1%	10.1(3.6–16.6)				NA			
≥ 1%	9.0 (5.8–12.2)				23.8 (12.9–34.7)			
PS score		0.811	–			0.408	–	
0–1	7.0 (1.5–8.5)				14.3 (11.8–16.9)			
= 2	6.0 (4.8–7.2)				14.6 (10.7–18.6)			
Stage		0.244	–			0.392	–	
III	5.0 (1.5–8.5)				10.0 (5.3–14.7)			
IVa	7.2 (4.6–9.8)				15.0 (13.0–17.0)			
IVb	9.0 (4.5–13.5)				15.0 (11.5–18.5)			
Chemotherapy drugs	9.8 (6.6–13.1)	0.041	0.434 (0.198–0.949)	0.036		0.276	0.499 (0.202–1.232)	0.132
containing pemetrexed	6.0 (5.1–6.9)				16.4 (9.9–22.9)			
containing paclitaxel	5.0 (3.2–6.8)				14.1 (11.5–16.7)			
Antiangiogenic therapy		< 0.001	0.307(0.117–0.805)	0.016		< 0.001	0.241(0.085–0.689)	0.008
Yes	9.8 (8.4–11.2)				20.0 (12.1–27.9)			
No	5.0 (3.2–6.8)				10.0 (6.5–13.5)			

In terms of the combination of chemotherapy and antiangiogenic therapy, TAX+AT group achieved the highest ORR and DCR compared to PEM + AT group (ORR:59.09% vs 30.56%, OR = 3.283, 95% CI 1.085–9.930, *P* = 0.032; DCR: 95.45% vs 91.67%, OR = 1.909, 95% CI 0.186–19.589, *P* = 0.985), PEM (no-AT) group (ORR:59.09% vs 12.5%, OR = 10.111, 95% CI 2.305–44.348, *P* = 0.001; DCR: 95.45% vs 66.67%, OR = 10.500, 95% CI 1.189–92.727, *P* = 0.037), and TAX (no-AT) group (ORR:59.09% vs 26.92%, OR = 3.921, 95% CI 1.165–13.198, *P* = 0.024; DCR: 95.45% vs 73.08%, OR = 7.737, 95% CI 0.870–68.803, *P* = 0.092). However, PEM + AT group had the longest PFS and OS compared to PEM (no-AT) group (PFS:14.0 vs 4.0 months, HR = 0.487, 95% CI 0.264–0.900, *P* = 0.009; OS: 25.0 vs 10.0 months, HR = 0.419, 95% CI 0.214–0.822, *P* = 0.006), TAX+AT group (PFS:14.0 vs 8.0 months, HR = 0.474, 95% CI 0.253–0.889, *P* = 0.008; OS: 25.0 vs 19.0 months, HR = 0.793, 95% CI 0.385–1.594, *P* = 0.508), and TAX (no-AT) group (PFS:14.0 vs 5.0 months, HR = 0.303, 95% CI 0.153–0.601, *P <* 0.001; OS: 25.0 vs 11.0 months, HR = 0.336, 95% CI 0.174–0.648, *P <* 0.001), as shown in Table 5, Fig. 6E and F.

**Table 5:** Treatment efficacy and prognosis of KRAS mutant NSCLC treated with different chemotherapy scheme

Treatment scheme	ORR	OR (95%CI)	*P* value	DCR	OR (95%CI)	*P* value
TAX+AT group	59.09%	–	–	95.45%	–	–
PEM + AT group	30.56%	3.283 (1.085–9.930)	0.032	91.67%	1.909 (0.186–19.589)	0.985
PEM (no-AT) group	12.50%	10.111 (2.305–44.348)	0.001	66.67%	10.500 (1.189–92.727)	0.037
TAX (no-AT) group	26.92%	3.921 (1.165–13.198)	0.024	73.08%	7.737 (0.870–68.803)	0.092
	Median PFS(95%CI)(month)	HR (95%CI)	*P* value	Median OS(95%CI)(month)	HR (95%CI)	*P* value
PEM + AT group	14.0 (9.9–18.1)	–	–	25.0 (5.7–44.3)	–	–
PEM (no-AT) group	4.0 (2.6–5.4)	0.487 (0.264–0.900)	0.009	10.0 (3.5–16.5)	0.419 (0.214–0.822)	0.006
TAX+AT group	8.0 (5.4–10.6)	0.474 (0.253–0.889)	0.008	19.0 (13.3–24.7)	0.793 (0.385–1.594)	0.508
TAX (no-AT) group	5.0 (3.7–6.3)	0.303 (0.153–0.601)	< 0.001	11.0 (6.11–15.9)	0.336 (0.174–0.648)	< 0.001

### Treatment efficacy of first-line immunotherapy

**Fig. 7:**
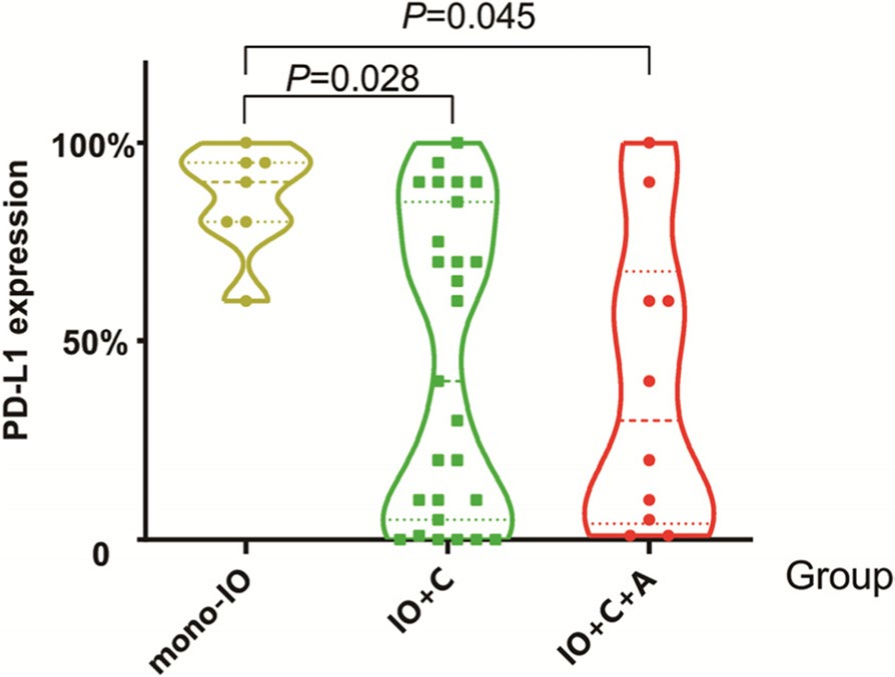
PD-L1 expression among the immunotherapy groups

We first analyzed the differences in PD-L1 expression among the immunotherapy groups, and found that mono-IO group has higher median PD-L1 expression compared to IO+C group, IO+C + A group (90.0% vs 40.0% vs 30.0%, *P* < 0.05, Fig. 7). Then we compared the treatment efficacy of above groups, no significant differences were found (ORR: 57.14% vs 42.86% vs 35.71%, *P* = 0.647; DCR: 85.71% vs 100.00% vs 92.86%, *P* = 0.152). Regarding the combined chemotherapy regimen, there were no statistical differences in ORR (50.00% vs 38.39%, *P* = 0.949) and DCR (100.00% vs 92.77.00%, *P* = 0.514) of immunotherapy combined paclitaxel-based or chemotherapy pemetrexed-based chemotherapy. In addition, patients with the common co-mutation TP53 did not show an efficacy difference in comparison to patients without TP53 (ORR: 53.33% vs 33.33%, *P* = 0.217; DCR: 100.00% vs 91.67%, *P* = 0.514).

## Discussion

KRAS was one of the frequent genes found to be mutated in NSCLC. Studies have shown that KRAS mutations account for about 5–15% of lung cancer in Asian patients, and about 20–30% non-Asian patients [21–23]. In our retrospective study, 9.86% of Chinese NSCLC patients harbor KRAS mutation, which was consistent with the results of previous study studies. LCMC study revealed that up to third of KRAS mutant lung adenocarcinomas patients harbored another oncogenic driver, the most common of which are TP53 and STK11 [22]. Our study supported TP53 was the prevalent co-mutations in KRAS mutant NSCLC, but STK11 was much less common. Several studies suggested that KRAS/TP53 co-mutation generally present with a significant upregulation of PD-L1 expression and tumoricidal T-cell accumulation, which may help NSCLC patients respond to immunotherapy and get long-term survival [24–27]. However, our study did not observe the correlation of KRAS/TP53 co-mutation and PD-L1 expression or immunotherapy efficacy. Although EGFR, KRAS, and ALK mutations are generally considered mutually exclusive in NSCLC [21], recent studies have shown that above oncogenic driver mutations can co-exist in a certain amount of lung cancers. A French study which includes 17,664 patients, identified 0.93% of non-squamous NSCLC with multiple genetic alterations involving oncogenic drivers, and the frequent concomitant mutations associated with KRAS included EGFR (15%) mutations [28]. Another south Korean study found the proportion of KRAS and EGFR co-mutation was 1.5% in KRAS mutant NSCLC patients [29]. We found in NSCLC patients, the co-mutation rate of EGFR and KRAS was 5.15%. In addition, it was reported that KRAS mutations in NSCLC mainly occurred in codons 12,13 or 61, and the most frequent subtypes were G12C, G12V and G12D [29, 30]. In this study, a total of 160 patients underwent KRAS subtypes testing, and the most common mutation sites of KRAS are located at codon 12, accounting for 82.5%, and KRAS G12C, G12V and G12D were the three most frequent subtypes, which was consistent with previous reports.

Although KRAS mutation is the first driver mutation to be discovered in solid tumors, this target has long been considered “undruggable” [31]. So KRAS mutated NSCLC patients have been treated as patients with driver mutation-negative patients, which currently take immunotherapy or chemotherapy as an available first-line therapeutic options. Studies demonstrated that TMB, PD-L1 expression is related to KRAS status in NSCLC [32, 33]. It is generally believed that KRASKRAS mutant NSCLC patients can benefit from immunotherapy. The subgroup analysis of CheckMate 057 study and OAK study indicated that during the second-line treatment for KRAS mutant NSCLC patients, immune checkpoint inhibitors (ICIs) monotherapy had a higher OS benefit than docetaxel monotherapy [34, 35]. In the first-line treatment, clinical trials have also shown ICIs could benefit KRAS mutant NSCLC patients. Subgroup analysis of KEYNOTE-042 study [36] showed that in patients with KRAS mutant NSCLC and a PD-L1 tumour proportion score (TPS) of 1% or greater, pembrolizumab monotherapy could significantly improve ORR (56.7% vs 18.0%), PFS (12 months vs. 6 months; HR = 0.51), OS (28 months vs. 11 months; HR = 0.42) compared with platinum-containing chemotherapy; and in patients with KRAS G12C mutant NSCLC, pembrolizumab monotherapy could also improve ORR (66.7% vs 23.5%), PFS (15 months vs. 6 months; HR = 0.27), OS (NR months vs. 8 months; HR = 0.28) compared with platinum-containing chemotherapy. Subgroup analysis of KEYNOTE-189 study [37] showed that pembrolizumab combined with pemetrexed-platinum has improved ORR (40.7% vs 26.7%), PFS (9 months vs. 5 months; HR = 0.47), OS (21 months vs. 14 months; HR = 0.79) compared with pemetrexed-platinum chemotherapy in KRAS mutant non-squamous NSCLC; in patients with KRAS G12C mutant non squamous NSCLC, ORR (50.0% vs. 18.2%), PFS (11 months vs. 5 months; HR = 0.48) were improved, but not OS (18 months vs. 25 months; HR = 1.14). Subgroup analysis of IMpower150 study [38] revealed that both the atezolizumab/bevacizumab/carboplatin/paclitaxel (ABCP) (PFS: 8.1, HR 0.42; OS: 19.8, HR 0.50) and ACP arms (PFS: 4.8, HR 0.80; OS: 11.7 months, HR 0.63) demonstrated survival improvements compared with the BCP arm (PFS: 5.8 months; OS: 9.9 months) in KRAS mutant NSCLC; Across PD-L1 subgroups in mKRAS patients, in high PD-L1 expression (≥50%) subgroup, a similar prolonged OS was observed for patients treated with both ABCP (23.9 months; HR 0.40) and ACP (median 19.9 months; HR 0.35) compared with BCP (median, 9.9 months) ;In low PD-L1 expression (1- < 50%) and PD-L1-negative(< 1%) subgroups, the OS in ABCP arm (17.5 and 22.4 months) were longer than BCP arm (4.8 and 7.9 months), but OS in ACP arm (5.0 and 8.7 months) was similar to BCP arm. The exploratory analysis results of the above three clinical studies showed that for NSCLC patients with KRAS or KRAS G12C mutation, immunotherapy alone or combined chemotherapy ± antiangiogenic therapy can bring better clinical benefits to patients than chemotherapy ± antiangiogenic therapy. Of course, the KRAS mutant NSCLC sample size of the three clinical studies is small, and there are differences in patient characteristics, and all patients in Checkmate 057 study has a PD-L1 tumour proportion score (TPS) of 1% or greater. In addition, patients in IMpower150 were stratified by PD-L1 subgroups , and the analysis result suggested the survival benefit of immunotherapy combined with chemotherapy is similar to that of chemotherapy alone in patients with low (1- < 50%) and negative(< 1%) PD-L1 expression, while immunotherapy combined with chemotherapy plus antiangiogenic therapy can achieve significant survival benefit in these patients. Our real-world data also found that immunotherapy brought significant survival benefits compared to chemotherapy in PD-L1 expression ≥1% subgroup. And antiangiogenic therapy could benefit KRAS mutant NSCLC patients, especially non-G12C KRAS subgroup. Unfortunately, due to the small sample size and immature survival data, we did not compare survival benefits of different regimens in PD-L1 expression < 1% subgroup. More studies are needed in the future to observe the immunotherapeutic efficacy of KRAS mutant NSCLC patients, especially in patients with PD-L1 expression < 1%.

Despite recent advances in NSCLC treatment, a number of patients still receive platinum-based chemotherapy as first-line therapy in real world. KRAS mutated NSCLC patients was considered to have low ORR and poor prognosis as a whole when receiving chemotherapy [39–41], thus appropriate selection of chemotherapy regimen is vital to improve the prognosis of those patients. In our retrospective study, the platinum-paclitaxel regimen has significantly higher ORR than platinum-pemetrexed regimen, but its PFS and OS were far inferior. Moreover, the addition of antiangiogenic therapy can significantly improve the ORR, PFS and OS in KRAS mutant NSCLC received chemotherapy as first-line treatment. In terms of the combination of chemotherapy and antiangiogenic therapy, platinum-paclitaxel combined with antiangiogenic therapy achieved the highest ORR and DCR, while platinum-pemetrexed combined with antiangiogenic therapy had the longest PFS and OS. Renaud S et al. [42] analyzed 1190 KRAS mutated stage IV NSCLC patients receiving first-line platinum-based chemotherapy and found that compared with pemetrexed and vinorelbine group, the paclitaxel group had the highest ORR (*P* < 0.001) and significantly improved TTP (P < 0.001). The ORR of the paclitaxel combined with bevacizumab group was higher (P < 0.001), which was consistent with the results of this study. Mellema WW et al. [43] retrospectively analyzed 464 KRAS mutated NSCLC patients, and found patients treated with taxanes had a significant improved ORR (50%) compared to pemetrexed (21%) or gemcitabine (25%; *P* < 0.01). Patients treated with bevacizumab in addition to taxanes had the highest ORR (62%). The PFS was significantly improved in patients treated with taxanes compared to pemetrexed (*P* = 0.02), but not OS (*P* = 0.41). On the contrary, there was retrospective studies [44] containing 75 KRAS mutated NSCLC patients found pemetrexed-based chemotherapy had higher efficacy than taxane-based chemotherapy. Our study comprehensively analyzed the efficacy and prognosis of different chemotherapy regimens for KRAS mutant NSCLC and found that platinum-pemetrexed regimen was more likely to provide survival benefits to patients, although platinum-paclitaxel regimen increased response rate. Moreover, the addition of antiangiogenic therapy can significantly improve both response rate and survival benefits. Okada F et al. [45] found that RAS-gene mutations could increase KRAS-dependent VEGF expression, promoting tumor angiogenesis and growth. T Konishi et al. [46] confirmed the K-ras gene regulated VEGF expression in NSCLC. The association between KRAS and VEGF may contribute to the response of KRAS mutant patients to antiangiogenic therapy. Our data supported platinum-pemetrexed combined with antiangiogenic therapy as the most effective treatment in patients with KRAS mutation NSCLC.

However, our study has limitation of single centre and retrospective nature. Our study included patients from 2014 to 2022, the large time span of the study may affect the consistency of the treatment plan, including the choice of chemotherapy and antiangiogenic drugs, follow-up treatment after first-line progress. In conclusion, this study shows that immunotherapy compared to chemotherapy, treatment containing pemetrexed compared to treatment containing paclitaxel, treatment with antiangiogenic therapy compared to those without could obtain more benefits for the first-line treatment of KRAS mutant NSCLC patients,. Subgroup analysis revealed the benefits of immunotherapy compared to chemotherapy were applicable PD-L1 expressio≥1% subgroup, and antiangiogenic therapy could benefit non-G12C KRAS subgroup, but not G12C KRAS subgroup.. Antiangiogenic therapy should be considered in the non-G12C KRAS mutant NSCLC patients on larger and prospective clinical trials. Given the improved survival benefits, platinum-pemetrexed combined with antiangiogenic therapy may be the preferred chemotherapy regimen for KRAS mutant NSCLC patients.

## Data Availability

The datasets used and/or analysed during the current study are available from the corresponding author on reasonable request.

## Abbreviations

NSCLC: Non-small cell lung cancer
KRAS: Kirsten rat sarcoma viral oncogene homolog
FDA: Food and drug administration
PS: Performance status
RECIST: Response evaluation criteria in solid tumors
CR: Complete response
PR: Partial response
SD: Stable disease
PD: Progressive disease
ORR: Objective response rate
DCR: Disease control rate
PFS: Progression-free survival
OS: Overall survival
OR: Odds ratio
95% CIs: 95% confidence intervals
HR: Hazard ratio
ICIs: Immune checkpoint inhibitors
